# Modeling Day-to-day Flow Dynamics on Degradable Transport Network

**DOI:** 10.1371/journal.pone.0168241

**Published:** 2016-12-13

**Authors:** Bo Gao, Ronghui Zhang, Xiaoming Lou

**Affiliations:** 1 Zhejiang Institute of Communications, Hangzhou, Zhejiang, P.R. China; 2 Research Center of Intelligent Transportation System, School of Engineering, Sun Yat-sen University, Guangzhou, Guangdong, P.R. China; 3 Energy Conversation and Emission Reduction Management Center of Zhejiang Provincial Communication Department, Hangzhou, Zhejiang, P.R. China; 4 School of Transportation, Southeast University, Nanjing, Jiangsu, P.R. China; Beihang University, CHINA

## Abstract

Stochastic link capacity degradations are common phenomena in transport network which can cause travel time variations and further can affect travelers’ daily route choice behaviors. This paper formulates a deterministic dynamic model, to capture the day-to-day (DTD) flow evolution process in the presence of degraded link capacity degradations. The aggregated network flow dynamics are driven by travelers’ study of uncertain travel time and their choice of risky routes. This paper applies the exponential-smoothing filter to describe travelers’ study of travel time variations, and meanwhile formulates risk attitude parameter updating equation to reflect travelers’ endogenous risk attitude evolution schema. In addition, this paper conducts theoretical analyses to investigate several significant mathematical characteristics implied in the proposed DTD model, including fixed point existence, uniqueness, stability and irreversibility. Numerical experiments are used to demonstrate the effectiveness of the DTD model and verify some important dynamic system properties.

## 1. Introduction

Day-to-day (DTD) traffic assignment model seems to be the most widely used approach in existing literatures to describe traveler’s individual route switching behavior, and the corresponding network traffic dynamic evolution at an aggregate level. Since the early work of Horowitz [1], the field has grown to potentially encompass a rather wide range of approaches, including deterministic processes and stochastic processes ([2–6]), and in the deterministic framework, these proposed processes can be divided into more detailed categories according to different equilibrium (or convergent) points, such as user equilibrium ([7–22]), stochastic user equilibrium ([1],[22–28]), partial user equilibrium [29] and bounded rational user equilibrium ([30,31]). Readers may refer to Cantarella [22] and Watling and [32] for both synthesis and development of the dynamic evolution process of traffic flows.

In existing DTD models, this adjustment process is usually demonstrated by two related traveler behavior mechanisms, including the experience learning mechanism and the route choice mechanism. In degradable transport network, travelers always suffer from within-day travel time uncertainties because of the intra-day link capacity degradations. In addition, travelers also experience day-to-day travel time variations caused by the inter-day fluctuation of traffic flow. The within-day and the day-to-day travel time variations together result in the route uncertainty or unreliability. In the context of route choice, the effect of route reliability is largely determined by traveler’s risk attitude. Therefore, for a realistic representation of the DTD traffic dynamics, it is essential to contain the integration of travel time uncertainty in the experience learning mechanism, and meanwhile account for travelers’ risk-taking behaviors in the route choice mechanism.

The DTD models previously introduced have addressed the integration of past experiences or other information sources to estimate the perceived mean travel time. However, they do not address the updating of travel time uncertainty, nor consider travelers’ risk-taking behaviors in the route choice processes. Jha *et al*. [33], Chen and Mahmassani [34] applied Bayesian learning model to complete the integration of travel time and its associated uncertainty. According to the learning rule governed by Bayesian theorem, these two studies only address the updating of the inherent within-day travel time uncertainty, but do not address the updating of day-to-day travel time uncertainty which is caused by the inter-day fluctuation of traffic flow. In addition, they have not considered travelers’ risk-taking behaviors in the context of route choice.

Risk attitudes can be captured by several theories such as prospect theory (PT) [35] or its cumulative representation (CPT) [36] and expected utility theory (EUT). In EUT, travelers are usually supposed to have exogenous risk attitudes which are reflected in the shape (concavity or convexity) of the utility function. This may be unreasonable because travelers’ past travel experiences are likely to influence their risk attitudes. In contrast with EUT, PT provides an implicit way to handle with the risk attitude evolution issue by updating the locations of reference points ([37–39]). Recently, some scholars (e.g. [40–42]) applied PT or CPT to examine the role of risk attitude in travelers’ DTD dynamic behaviors. Their models are potential to provide well-supported descriptive paradigm for decision making under uncertainty, but at the same time, these models adopt a quite large number of behavioral parameters which may lead to the difficulty of model calibration and validation.

The main objective of this paper is to describe the aggregate network flow DTD dynamics by considering both travelers’ study of uncertain travel time and their choice of risky routes. This work is mainly inspired by the objective reality that uncertainties often exist in traffic systems because of the inter-day traffic flow variations and the intra-day road capacity degradations. With the presented model, this paper also makes some efforts to examine the effects of travel time uncertainties and travelers’ risk attitudes on traffic flow evolution and other dynamic system properties, particularly convergence, stability and irreversibility.

In the presented DTD model, the notion of variation range is adopted to indicate travel time uncertainty information. Mathematically, this notion is expressed as the difference between the longest and the shortest travel time values. In contrast with the traditional travel time variance (or its distribution), variation range seems to be a more reasonable indicator reflecting travel time uncertainty because in the real world travelers appear more sensitive to the extreme travel time value (e.g. the longest or shortest one) than to the specific travel time distribution. In addition, in the proposed model, an endogenous risk attitude evolution schema is given to reflect that travelers constantly adjust their risk attitudes through learning their past travel experiences.

This study advances previous work by making the following specific contributions. First, a simple but effective indicator about travel time, namely variation range, is used to indicate its uncertainty information. Second, the within-day and the day-to-day travel time variation ranges, which are respectively caused by the intra-day road capacity degradations and the inter-day traffic flow fluctuation, are both considered to reflect traveler’s sense of route unreliability or risk. Third, an endogenous risk attitude evolution schema is adopted to reflect the change of traveler’s risk attitude in the context of day-to-day traffic evolution. Finally, the above ideas are integrated into a DTD model to examine their effects on the whole day-to-day behavior of traffic flows.

In the next section, a new DTD model, which contains both travelers’ study of uncertain travel time and their choices towards risky travel route, is proposed to describe the realistic dynamic traffic flow evolution process. In Section 3, some theoretical analyses are conducted to investigate the mathematical properties implied in the proposed DTD model. Section 4 applies the proposed model to a test network to demonstrate the effectiveness of the model and verify some important dynamic system properties such as convergence, stability and irreversibility. Section 5 concludes the paper.

## 2. Description of the DTD Model

### 2.1 Degradable Traffic Network and Relevant Notions Definition

A traffic network is a directed graph (*N*,*L*) where *N* represents the node set and *L* corresponds to the link or road set. The notions that will be used in this paper are listed in Table 1.

**Table 1:** Notions and the corresponding definitions.

Notion	Definition
*l* ∈ *L*	link index;
*O*	origin set, *o* ∈ *O*, *O* ⊆ *N*;
*D*	destination set, *d* ∈ *D*, *D* ⊆ *N*;
*R*_*od*_	route set between OD pair (*o*,*d*);
*r* ∈ *R*_*od*_	route index;
*q*_*od*_	travel demand on OD pair (*o*,*d*);
$x_{l}^{t}$	flow on link *l* on day *t*;
$f_{r}^{t}$	flow on route *r* on day *t*;
$u_{l}^{t}$	stochastic traffic capacity of link *l* on day *t*, its value varies from the lower limit ${\underline{u}}_{l}^{t}$ to the upper limit ${\bar{u}}_{l}^{t}$, $u_{l}^{t}\in [{\underline{u}}_{l}^{t},{\bar{u}}_{l}^{t}]$;
${\dot{u}}_{l}^{t}$	mean capacity of link *l* on day *t*;
$c_{l}(x_{l}^{t},u_{l}^{t})$	stochastic travel time of link *l* on day *t*, as a function of flow $x_{l}^{t}$ and stochastic capacity $u_{l}^{t}$;
$c_{l}(x_{l}^{t},{\bar{u}}_{l}^{t})$	lower limit of within-day link travel time, as a function of flow $x_{l}^{t}$ and maximum capacity ${\bar{u}}_{l}^{t}$;
$c_{l}(x_{l}^{t},{\underline{u}}_{l}^{t})$	upper limit of within-day link travel time, as a function of flow $x_{l}^{t}$ and minimum capacity ${\underline{u}}_{l}^{t}$;
$c_{l}(x_{l}^{t},{\dot{u}}_{l}^{t})$	actual mean travel time of link *l* on day *t*, as a function of flow $x_{l}^{t}$ and mean capacity ${\dot{u}}_{l}^{t}$;
$\delta c_{l}^{t}$	actual within-day variation range of link travel time;
Λ_*lr*_	link-route index; if route *r* traverses link *l*, Λ_*lr*_ = 1, otherwise, Λ_*lr*_ = 0;
$S_{r}^{t}$	stochastic travel time of route *r* on day *t*, $S_{r}^{t}=\sum_{l}\Lambda_{lr}∙c_{l}(x_{l}^{t},u_{l}^{t})$;
${\underline{S}}_{r}^{t}$	lower limit of within-day route travel time, ${\underline{S}}_{r}^{t}=\sum_{l}\Lambda_{lr}∙c_{l}(x_{l}^{t},{\bar{u}}_{l}^{t})$;
${\bar{S}}_{r}^{t}$	upper limit of within-day route travel time, ${\bar{S}}_{r}^{t}=\sum_{l}\Lambda_{lr}∙c_{l}(x_{l}^{t},{\underline{u}}_{l}^{t})$;
${\dot{S}}_{r}^{t}$	actual mean travel time of route *r* on day *t*, ${\dot{S}}_{r}^{t}=\sum_{l}\Lambda_{lr}∙c_{l}(x_{l}^{t},{\dot{u}}_{l}^{t})$;
${\tilde{S}}_{r}^{t}$	perceived mean travel time of route *r* on day *t*;
$\delta S_{r}^{t}$	actual within-day variation range of route travel time, $\delta S_{r}^{t}={\bar{S}}_{r}^{t}-{\underline{S}}_{r}^{t}$;
$\delta {\tilde{S}}_{r}^{t}$	perceived within-day variation range of route travel time;
$\Delta S_{r}^{t}$	actual day-to-day variation range of route travel time;
$\Delta {\tilde{S}}_{r}^{t}$	perceived day-to-day variation range of route travel time;
$\rho_{od}^{t}$	risk attitude parameter for traveling between OD pair (*o*,*d*) on day *t*, its value range is set as [−*ρ*_max_,*ρ*_max_], *ρ*_max_ > 0;
$h_{r}^{t}$	systematic disutility value associated to route *r* on day *t*;
$P_{rod}^{t}$	the probability travelers choose path *r* ∈ *R*_*od*_ on day *t*.

Some other parameters used in the proposed DTD model will be defined when first introduced.

### 2.2 Travelers’ Perceptions of Route Travel Time and Its Variation Ranges

Following the previous works (e.g. [1,4,22,23,26–28]), travelers’ perceptions of mean route travel time are built up through an exponential-smoothing style of learning process, which involves a weighted combination of the perceived and actual mean time on the previous days. This learning process can be represented by the following recursion equation:

$${\tilde{S}}_{r}^{t+1}=\alpha ∙{\dot{S}}_{r}^{t}+(1-\alpha )∙{\tilde{S}}_{r}^{t}=\alpha ∙\sum_{l\in L}\Lambda_{lr}∙c_{l}(x_{l}^{t},{\dot{u}}_{l}^{t})+(1-\alpha )∙{\tilde{S}}_{r}^{t},\;\forall r\in R_{od},$$ (1)

where *α*(0 ≤ *α* ≤ 1) denotes a constant parameter (independent of *t*), which reflects travelers’ preference between actual and expected route travel time.

Actually, the exponential-smoothing filter and the preference parameter *α* together reflect travelers’ learning mechanisms and memory characteristics about the past experiences, they may apply not only to the perceptions of mean route travel time, but also to the perceptions of travel time uncertainties. Therefore, this study uses the same exponential-smoothing filter and preference parameter to establish the updating equation of route travel time variation ranges in the following content.

For the day-to-day research framework as considered in our paper, the within-day flow dynamics of the traffic system is usually neglected in existing literatures. The day-to-day dynamic model only considers the flow evolution process along the large-scale time scale ‘day’. The within-day realization process, on the other hand, mainly address the real-time dynamic traffic flow as the realization of the travellers’ route choices on a particular day, which, in turn, results in updated information feedback to the day-to-day process. Until now, most works investigate these two dynamical processes independently, and some attempts (e.g. [43–47]) are still on the road to combine these two problems into a unified doubly dynamic traffic assignment model.

In this paper, we adopt the within-day flow static assumption to eliminate the effects of within-day flow dynamics on travel time variations, and focus only on the day-to-day research framework. The within-day static assumption allows a mathematical formalization that is easier to manage in theoretical terms. From this point of view, the only cause leading to within-day link travel time variations $\left(\delta c_{l}^{t}\right)$ is the intra-day link capacity fluctuations. Mathematically, $\delta c_{l}^{t}$ can be expressed as the difference between $c_{l}(x_{l}^{t},{\underline{u}}_{l}^{t})$ and $c_{l}(x_{l}^{t},{\bar{u}}_{l}^{t})$:

$$\delta c_{l}^{t}=c_{l}(x_{l}^{t},{\underline{u}}_{l}^{t})-c_{l}(x_{l}^{t},{\bar{u}}_{l}^{t}),\;\forall l\in L.$$ (2)

The actual variation range of within-day route travel time, namely $\delta S_{r}^{t}$, can then be obtained immediately according to the link-route topological relation:

$$\begin{array}{l} \delta S_{r}^{t}={\bar{S}}_{r}^{t}-{\underline{S}}_{r}^{t}=\sum_{l}\Lambda_{lr}∙c_{l}(x_{l}^{t},{\underline{u}}_{l}^{t})-\sum_{l}\Lambda_{lr}∙c_{l}(x_{l}^{t},{\bar{u}}_{l}^{t})\\ \;=\sum_{l}\Lambda_{lr}∙(c_{l}(x_{l}^{t},{\underline{u}}_{l}^{t})-c_{l}(x_{l}^{t},{\bar{u}}_{l}^{t}))=\sum_{l}\Lambda_{lr}∙\delta c_{l}^{t},\;\forall r\in R_{od}. \end{array}$$ (3)

Then the perceived variation range $\left(\delta {\tilde{S}}_{r}^{t+1}\right)$ can be achieved by applying the exponential-smoothing updating process:

$$\delta {\tilde{S}}_{r}^{t+1}=\alpha ∙\delta S_{r}^{t}+(1-\alpha )∙\delta {\tilde{S}}_{r}^{t}=\alpha ∙\sum_{l}\Lambda_{lr}∙\delta c_{l}^{t}+(1-\alpha )∙\delta {\tilde{S}}_{r}^{t},\;\forall r\in R_{od}.$$ (4)

Besides of the within-day variation $\delta S_{r}^{t}$, travelers will also perceive day-to-day travel time fluctuation $\left(\Delta S_{r}^{t}\right)$ from their past experiences. Mathematically, $\Delta S_{r}^{t}$ can be represented as the difference of the mean route travel time on two consecutive days:

$$\Delta S_{r}^{t}=|{\dot{S}}_{r}^{t}-{\dot{S}}_{r}^{t-1}|=|\sum_{l}\Lambda_{lr}∙c_{l}(x_{l}^{t},{\dot{u}}_{l}^{t})-\sum_{l}\Lambda_{lr}∙c_{l}(x_{l}^{t-1},{\dot{u}}_{l}^{t-1})|,\;\forall r\in R_{od}.$$ (5)

In Eq 5, the absolute value sign |∙| is applied to guarantee the non-negativity of $\Delta S_{r}^{t}$. Note that the effect of intra-day link capacity fluctuation is not reflected in $\Delta S_{r}^{t}$ because it has been considered by the within-day variation $\delta S_{r}^{t}$.

Then the perceived day-to-day variation range can be updated still through the exponential-smoothing type recursion equation:

$$\Delta {\tilde{S}}_{r}^{t+1}=\alpha ∙|\sum_{l}\Lambda_{lr}∙c_{l}(x_{l}^{t},{\dot{u}}_{l}^{t})-\sum_{l}\Lambda_{lr}∙c_{l}(x_{l}^{t-1},{\dot{u}}_{l}^{t-1})|+(1-\alpha )∙\Delta {\tilde{S}}_{r}^{t},\;\forall r\in R_{od}.$$ (6)

$\delta {\tilde{S}}_{r}^{t+1}$ and $\Delta {\tilde{S}}_{r}^{t+1}$ together present the integrated description of travel time uncertainties. In this paper, the integrated travel time variation range is defined as the sum of $\delta {\tilde{S}}_{r}^{t+1}$ and $\Delta {\tilde{S}}_{r}^{t+1}$, its updating equation can be easily achieved by combining Eqs 4 and 6 together:

$$\delta {\tilde{S}}_{r}^{t+1}+\Delta {\tilde{S}}_{r}^{t+1}=\alpha ∙(\delta S_{r}^{t}+\Delta S_{r}^{t})+(1-\alpha )∙(\delta {\tilde{S}}_{r}^{t}+\Delta {\tilde{S}}_{r}^{t})=\alpha ∙(\sum_{l}\Lambda_{lr}∙\delta c_{l}^{t}+|\sum_{l}\Lambda_{lr}∙c_{l}(x_{l}^{t},{\dot{u}}_{l}^{t})-\sum_{l}\Lambda_{lr}∙c_{l}(x_{l}^{t-1},{\dot{u}}_{l}^{t-1})|)+(1-\alpha )∙(\delta {\tilde{S}}_{r}^{t}+\Delta {\tilde{S}}_{r}^{t}),\;\forall r\in R_{od}.$$ (7)

### 2.3 Travelers’ Perception of Systematic Disutility Associated to Every Route

To model travelers’ route choice and adjustment, the key is to calculate the systematic disutility of every alternative route. Traditionally, the systematic disutility is usually defined as an affine transformation of the mean route travel time without consideration of travel time variations. In this section, the traditional disutility representation is modified to reflect the effect of travel time uncertainty. This modification is derived under some mild assumptions as stated below.

**Assumption I**: All the possible values of the perceived route travel time are distributed continuously in an interval whose length is defined by the integrated variation range $\left(\delta {\tilde{S}}_{r}^{t+1}+\Delta {\tilde{S}}_{r}^{t+1}\right)$; the perceived mean route travel time $\left({\tilde{S}}_{r}^{t+1}\right)$ is located at the middle point of this interval, and the other route travel time are distributed symmetrically on the left and right sides of ${\tilde{S}}_{r}^{t+1}$.

**Assumption II**: If the integrated variation range is zero, then the systematic disutility $\left(h_{r}^{t+1}\right)$ is equal to the perceived mean route travel time $\left({\tilde{S}}_{r}^{t+1}\right)$.

For ease of description, we define *η*(*x*) and *μ*(*x*), respectively, to represent the disutility function and the probability density function (PDF) of the perceived route travel time *x*. Assumption reveals’ the following conditions:

$$x\in \left[{\tilde{S}}_{r}^{t+1}-0.5(\delta {\tilde{S}}_{r}^{t+1}+\Delta {\tilde{S}}_{r}^{t+1}),{\tilde{S}}_{r}^{t+1}+0.5(\delta {\tilde{S}}_{r}^{t+1}+\Delta {\tilde{S}}_{r}^{t+1})\right],$$

$\mu (x)=\mu (2{\tilde{S}}_{r}^{t+1}-x)$ and $\partial \mu (x)/\partial x=-\partial \mu (2{\tilde{S}}_{r}^{t+1}-x)/\partial x$ (the symmetrical distribution).

In addition, according to the nature of PDF, if $\delta {\tilde{S}}_{r}^{t+1}+\Delta {\tilde{S}}_{r}^{t+1}=0$, then $h_{r}^{t+1}={\tilde{S}}_{r}^{t+1}$, which actually presents an anchor point of the applied disutility function, namely $\eta ({\tilde{S}}_{r}^{t+1})={\tilde{S}}_{r}^{t+1}$. On the other hand, if $\delta {\tilde{S}}_{r}^{t+1}+\Delta {\tilde{S}}_{r}^{t+1}>0$, the systematic disutility $h_{r}^{t+1}$ can be represented as follows:

$$\begin{array}{l} h_{r}^{t+1}=\int_{{\tilde{S}}_{r}^{t+1}-0.5\left(\delta {\tilde{S}}_{r}^{t+1}+\Delta {\tilde{S}}_{r}^{t+1}\right)}^{{\tilde{S}}_{r}^{t+1}+0.5\left(\delta {\tilde{S}}_{r}^{t+1}+\Delta {\tilde{S}}_{r}^{t+1}\right)}\eta \left(x\right)∙\mu \left(x\right)dx\\ \;=\int_{{\tilde{S}}_{r}^{t+1}-0.5\left(\delta {\tilde{S}}_{r}^{t+1}+\Delta {\tilde{S}}_{r}^{t+1}\right)}^{{\tilde{S}}_{r}^{t+1}}\eta \left(x\right)∙\mu \left(x\right)dx+\int_{{\tilde{S}}_{r}^{t+1}}^{{\tilde{S}}_{r}^{t+1}+0.5\left(\delta {\tilde{S}}_{r}^{t+1}+\Delta {\tilde{S}}_{r}^{t+1}\right)}\eta \left(x\right)∙\mu \left(x\right)dx\\ \;=\int_{{\tilde{S}}_{r}^{t+1}-0.5\left(\delta {\tilde{S}}_{r}^{t+1}+\Delta {\tilde{S}}_{r}^{t+1}\right)}^{{\tilde{S}}_{r}^{t+1}}\eta \left(x\right)∙\mu \left(x\right)dx-\int_{{\tilde{S}}_{r}^{t+1}}^{{\tilde{S}}_{r}^{t+1}-0.5\left(\delta {\tilde{S}}_{r}^{t+1}+\Delta {\tilde{S}}_{r}^{t+1}\right)}\eta \left(2{\tilde{S}}_{r}^{t+1}-x\right)∙\mu \left(2{\tilde{S}}_{r}^{t+1}-x\right)dx\\ \;=\int_{{\tilde{S}}_{r}^{t+1}-0.5\left(\delta {\tilde{S}}_{r}^{t+1}+\Delta {\tilde{S}}_{r}^{t+1}\right)}^{{\tilde{S}}_{r}^{t+1}}\left(\eta \left(x\right)+\eta \left(2{\tilde{S}}_{r}^{t+1}-x\right)\right)∙\mu \left(x\right)dx,\;\forall r\in R_{od}. \end{array}$$ (8)

Although the value of $h_{r}^{t+1}$ cannot be achieved from Eq 8 since the specific formulations of *η*(*x*) and *μ*(*x*) are both not given in this study, however, some fundamental properties implied in $h_{r}^{t+1}$ can still be analyzed by considering the conditions given by Assumptions I and II as well as travelers’ risk attitudes. These properties are essential to establish simplified expression and then to calculate approximate value of the systematic disutility $h_{r}^{t+1}$.

Consider the first situation in which travelers are assumed to be risk averse. According to EUT, risk aversion is associated with a convex disutility function. The convexity implies the following inequality:

$$\eta (x)+\eta (2{\tilde{S}}_{r}^{t+1}-x)>2{\tilde{S}}_{r}^{t+1},\;(x\neq {\tilde{S}}_{r}^{t+1}).$$

With this inequality, the systematic disutility $h_{r}^{t+1}$ given by Eq 8 can be compared with the perceived mean route travel time $\left({\tilde{S}}_{r}^{t+1}\right)$ as follows:

$$h_{r}^{t+1}=\int_{{\tilde{S}}_{r}^{t+1}-0.5(\delta {\tilde{S}}_{r}^{t+1}+\Delta {\tilde{S}}_{r}^{t+1})}^{{\tilde{S}}_{r}^{t+1}}(\eta (x)+\eta (2{\tilde{S}}_{r}^{t+1}-x))∙\mu (x)dx>{\tilde{S}}_{r}^{t+1},\;\forall r\in R_{od}.$$ (9)

In addition, when $x\in \left({\tilde{S}}_{r}^{t+1}-0.5(\delta {\tilde{S}}_{r}^{t+1}+\Delta {\tilde{S}}_{r}^{t+1}),{\tilde{S}}_{r}^{t+1}\right)$, the convexity of the disutility function also implies the increase velocity of $\eta (2{\tilde{S}}_{r}^{t+1}-x)$ towards its right side is larger than the decrease velocity of *η*(*x*) towards the left side.This means when the integrated variation range $\left(\delta {\tilde{S}}_{r}^{t+1}+\Delta {\tilde{S}}_{r}^{t+1}\right)$ increases, the systematic disutility $h_{r}^{t+1}$ perceived by travelers will increase simultaneously. Through the above analysis, we can find the first property implied in the systematic disutility which is associated with risk aversion. This property is stated as below.

**Property I**: Under the conditions of Assumptions I and II, if travelers are assumed to be risk averse, then their perceived systematic disutility has a larger value than the perceived mean route travel time, this disutility is positively associated with their perceived travel time variation ranges.

Besides of risk aversion, risk proneness and risk neutrality can also be reflected in EUT, which correspond respectively to concave and linear disutility functions. In both situations, the previous analysis can still be carried out to study the other properties of the systematic disutility. These properties are summarized as below.

**Property II**: Under the conditions of Assumptions I and II, if travelers are risk prone, then their perceived systematic disutility has a smaller value than the perceived mean route travel time, which is negatively associated with the perceived travel time variation ranges.

**Property III**: Under the conditions of Assumptions I and II, if travelers are risk neutral, then their perceived systematic disutility is equal to the perceived mean route travel time, regardless of their perceived travel time variation ranges.

After acquiring Properties I~III, it is time now to establish a simplified expression of $h_{r}^{t+1}$ reflecting travelers’ perception of route systematic disutility. In this study, $h_{r}^{t+1}$ is formulated as the weighted sum of the perceived mean route travel time and its associated variation ranges:

$$h_{r}^{t+1}={\tilde{S}}_{r}^{t+1}+\rho_{od}^{t+1}∙(\delta {\tilde{S}}_{r}^{t+1}+\Delta {\tilde{S}}_{r}^{t+1}),\;\forall r\in R_{od},$$ (10)

Where $\rho_{od}^{t+1}$ is defined as the risk attitude parameter, its value range is set as [−*ρ*_max_,*ρ*_max_]. According to Properties I~III, a positive value of $\rho_{od}^{t+1}$ indicates risk aversion, while risk proneness is associated with a negative $\rho_{od}^{t+1}$, and when travelers are risk neutral, $\rho_{od}^{t+1}$ is equal to zero.

In reality, different travelers always have different risk attitudes, this suggests the risk attitude parameter should be defined at the individual level. However, defining specific risk attitude for every traveler is a tedious work, which may greatly hinder the execution efficiency of the proposed DTD model. The focus of this paper is not to study traveler’s individual behavior, but to investigate the network traffic dynamic evolution at an aggregate level. Therefore, the unified parameter $\rho_{od}^{t+1}$ in Eq 10 can be treated as an aggregated form of the realistic travelers’ risk attitudes.

### 2.4 The Evolution of Travelers’ Risk Attitudes

Most of the existing studies concerning risk-taking behaviors focus only on static decision scenario in which travelers’ risk attitudes are exogenous and changeless, the suitability extension to consider endogenous risk attitude evolution in the dynamical or time-varying environment is quite lacking. Barkan and Busemeyer [37] examined decision makers’ risk attitude change in a sequential two-gamble scenario, and found risk prone after an anticipated loss while risk aversion after an anticipated gain, which could be explained by the reference point changes in PT or CPT.

**Fig 1:**
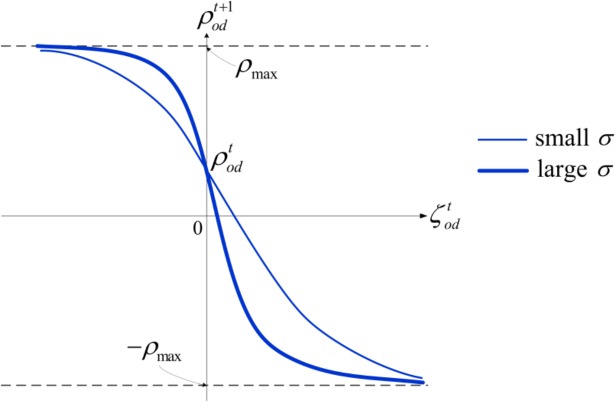
Updatings of the risk attitude parameter $\rho_{od}^{t+1}$ with different values of parameter *σ*. A larger *σ*-value corresponds to a larger change rate of $\rho_{od}^{t}$.

In the DTD traffic dynamics, travelers can get their travel time saving (namely travel gain), if the actual travel time cost they experienced on the current day is shorter than the perceived travel time (reference point) they estimated on the previous day. As a result, these travelers will show risk attitude change trend towards risk aversion. Conversely, travelers learn loss if the experienced travel time is longer than the perceived one, and in this situation, travelers will behave attitude change towards risk proneness. According to this rule, an updating equation about the risk attitude parameter $\left(\rho_{od}^{t}\right)$ is proposed to reflect the endogenous risk attitude evolution schema:

$$\rho_{od}^{t+1}=\frac{2\rho_{max}∙(\rho_{max}+\rho_{od}^{t})}{\rho_{max}+\rho_{od}^{t}+(\rho_{max}-\rho_{od}^{t})∙exp\{\sigma ∙\zeta_{od}^{t}\}}-\rho_{max},$$ (11)

where $\zeta_{od}^{t}$ is defined to represent travel time saving or losing perceived by travelers on day *t*, its value is calculated as $\left(\sum_{r\in R_{od}}{\dot{S}}_{r}^{t}∙f_{r}^{t}-\sum_{r\in R_{od}}{\tilde{S}}_{r}^{t}∙f_{r}^{t-1}\right)/q_{od}$. The constant parameter *σ*(*σ* ≥ 0) in Eq 11 is defined to represent travelers’ sensitivity to their travel time saving or losing. The updating process of $\rho_{od}^{t+1}$ defined by Eq 11 is depicted in Fig 1.

In Fig 1, all possible values of $\rho_{od}^{t+1}$ are located in the range of −*ρ*_max_ to *ρ*_max_. When $\zeta_{od}^{t}<0$ (in the domain of gain), traveler’s risk attitude act out evolution trend towards risk aversion, which results in the increase of the risk attitude parameter, namely $\rho_{od}^{t+1}>\rho_{od}^{t}$. On the contrary, when $\zeta_{od}^{t}>0$ (in the domain of loss), travelers behave attitude change towards risk proneness, which leads to the risk attitude parameter decrease $\left(\rho_{od}^{t+1}<\rho_{od}^{t}\right)$.

### 2.5 The Evolution of Link Traffic Flow

In reality, because of traffic congestion and perception deviation, some random residuals are introduced into the systematic disutility $h_{r}^{t+1}$ to influence travelers’ route choices. If the random residuals are assumed to be independent over time scales, OD pairs and routes, and furthermore, if they are identically distributed as Gumbel random variables with zero mean, then travelers’ route choice probability can be given by a Logit model:

$$P_{rod}^{t+1}=\frac{exp\{-\theta ∙h_{r}^{t+1}\}}{\sum_{k\in R_{od}}exp\{-\theta ∙h_{k}^{t+1}\}},\;\forall r\in R_{od}.$$ (12)

The positive dispersion parameter *θ* in Eq 12 reflects the degree of familiarity with conditions by travelers, a higher *θ*-value means a smaller perception variation.

With travelers’ route choice probability, the evolution of link traffic flow can then be formulated by considering inertial travelers:

$$x_{l}^{t+1}=\beta ∙\sum_{o\in O}\sum_{d\in D}\sum_{r\in R_{od}}\Lambda_{lr}∙q_{od}∙\frac{exp\{-\theta ∙h_{r}^{t+1}\}}{\sum_{k\in R_{od}}exp\{-\theta ∙h_{k}^{t+1}\}}+(1-\beta )∙x_{l}^{t},\;\forall l\in L.$$ (13)

The inertial parameter *β*(0 < *β* < 1) indicates the proportion of travelers reconsidering the previous choice.

## 3. Theoretical Analysis of the Proposed DTD Model

This section provides some theoretical analysis to investigate several important properties implied in the proposed dynamic system. To facilitate the analysis, the DTD model presented in Section 2 is reformulated as below.

$$\begin{array}{c} {\tilde{S}}_{r}^{t+1}=\alpha ∙\sum_{l\in L}\Lambda_{lr}∙c_{l}\left(x_{l}^{t},{\dot{u}}_{l}^{t}\right)+\left(1-\alpha \right)∙{\tilde{S}}_{r}^{t},\;\forall r\in R_{od},\\ \delta {\tilde{S}}_{r}^{t+1}=\alpha ∙\sum_{l}\Lambda_{lr}∙\left(c_{l}\left(x_{l}^{t},{\underline{u}}_{l}^{t}\right)-c_{l}\left(x_{l}^{t},{\bar{u}}_{l}^{t}\right)\right)+\left(1-\alpha \right)∙\delta {\tilde{S}}_{r}^{t},\;\forall r\in R_{od},\\ \Delta {\tilde{S}}_{r}^{t+1}=\alpha ∙\left|\sum_{l}\Lambda_{lr}∙c_{l}\left(x_{l}^{t},{\dot{u}}_{l}^{t}\right)-\sum_{l}\Lambda_{lr}∙c_{l}\left(x_{l}^{t-1},{\dot{u}}_{l}^{t-1}\right)\right|+\left(1-\alpha \right)∙\Delta {\tilde{S}}_{r}^{t},\;\forall r\in R_{od},\\ \rho_{od}^{t+1}=\frac{2\rho_{\max}∙\left(\rho_{\max}+\rho_{od}^{t}\right)}{\rho_{\max}+\rho_{od}^{t}+\left(\rho_{\max}-\rho_{od}^{t}\right)∙\exp\left\{\sigma ∙\zeta_{od}^{t}\right\}}-\rho_{\max},\\ h_{r}^{t+1}={\tilde{S}}_{r}^{t+1}+\rho_{od}^{t+1}∙\left(\delta {\tilde{S}}_{r}^{t+1}+\Delta {\tilde{S}}_{r}^{t+1}\right),\;\forall r\in R_{od},\\ x_{l}^{t+1}=\beta ∙\sum_{o\in O}\sum_{d\in D}\sum_{r\in R_{od}}\Lambda_{lr}∙q_{od}∙\frac{\exp\left\{-\theta ∙h_{r}^{t+1}\right\}}{\sum_{k\in R_{od}}\exp\left\{-\theta ∙h_{k}^{t+1}\right\}}+\left(1-\beta \right)∙x_{l}^{t},\;\forall l\in L. \end{array}\}$$ (14)

### 3.1 Fixed Point (FP) of the DTD Evolution Process and Equilibrium State

FP of the DTD dynamic process (14) is obtained from conditions ${\tilde{S}}_{r}^{t+1}={\tilde{S}}_{r}^{t}={\tilde{S}}_{r}^{*}$, $\delta {\tilde{S}}_{r}^{t+1}=\delta {\tilde{S}}_{r}^{t}=\delta {\tilde{S}}_{r}^{*}$, $\Delta {\tilde{S}}_{r}^{t+1}=\Delta {\tilde{S}}_{r}^{t}=\Delta {\tilde{S}}_{r}^{*}$, $\rho_{od}^{t+1}=\rho_{od}^{t}=\rho_{od}^{*}$ and $x_{l}^{t+1}=x_{l}^{t}=x_{l}^{*}$, thus:

$${\tilde{S}}_{r}^{*}=\sum_{l}\Lambda_{lr}∙c_{l}(x_{l}^{*},{\dot{u}}_{l}^{t}),\;\forall r\in R_{od},$$ (15)

$${\tilde{S}}_{r}^{*}=\sum_{l}\Lambda_{lr}∙(c_{l}(x_{l}^{*},{\underline{u}}_{l}^{t})-c_{l}(x_{l}^{*},{\bar{u}}_{l}^{t})),\;\forall r\in R_{od},$$ (16)

$$\Delta {\tilde{S}}_{r}^{*}=|\sum_{l}\Lambda_{lr}∙c_{l}(x_{l}^{*},{\dot{u}}_{l}^{t})-\sum_{l}\Lambda_{lr}∙c_{l}(x_{l}^{*},{\dot{u}}_{l}^{t-1})|,\;\forall r\in R_{od},$$ (17)

$$h_{r}^{*}={\tilde{S}}_{r}^{*}+\rho_{od}^{*}∙(\delta {\tilde{S}}_{r}^{*}+\Delta {\tilde{S}}_{r}^{*}),\;\forall r\in R_{od},$$ (18)

$$x_{l}^{*}=\sum_{o\in O}\sum_{d\in D}\sum_{r\in R_{od}}\Lambda_{lr}∙q_{od}∙\frac{exp\{-\theta ∙h_{r}^{*}\}}{\sum_{k\in R_{od}}exp\{-\theta ∙h_{k}^{*}\}},\;\forall l\in L.$$ (19)

Obviously, FP described by Eq 19 is equivalent to the well-known SUE state, which is extensively reviewed in the literatures. It is worth noting that, fixed-point attractor of the proposed dynamic process not only depend upon the route systematic disutility $h_{r}^{*}$ but also upon the route choice behavior and the various kind of parameters (including *α*, *β*, *σ* and *θ*) adopted in this model.

The implicit relation between model parameters *α*, *β* and fixed-point attractor of the proposed dynamic process, in fact, is determined by the risk attitude evolution schema $\left(\rho_{od}^{t+1}\right)$ defined by Eq 11. This schema indicates that the endogenous risk attitude is influenced by travelers’ perceived travel time saving $\zeta_{od}^{t}$, which is further influenced by the model parameters *α* and *β* since the value of $\zeta_{od}^{t}$ is calculated as $\left(\sum_{r\in R_{od}}{\dot{S}}_{r}^{t}∙f_{r}^{t}-\sum_{r\in R_{od}}{\tilde{S}}_{r}^{t}∙f_{r}^{t-1}\right)/q_{od}$.

Furthermore, the evolution of $\rho_{od}^{t}$ defined by Eq 11 is an irreversible process (see Section 3.4) which means the latest risk attitude parameter $\rho_{od}^{t+1}$ will change under any small fluctuation of network traffic flow state. Under this situation, the stable risk attitude $\rho_{od}^{*}$ in Eq 18 is not fixed, it is determined by the past system evolution process and therefore is influenced by parameters *α* and *β*. The risk attitude evolution schema (Eq 11) also implies the stable risk attitude $\rho_{od}^{*}$ is influenced by parameter *σ*. In addition, the Logit model defines the effect of parameter *θ* on the ultimate route choice results. As a consequence, fixed-point attractor of the proposed dynamic process is influenced together by the model parameters *α*, *β*, *σ* and *θ*.

### 3.2 FP Existence

Sufficient conditions for fixed-point existence can be easily derived through Brouwer’s fixed point theorem, requiring continuity of all involved functions. Note that fixed-point condition presented by Eq 19 actually defines a map of $x_{l}^{*}$ to itself. Then fixed-point existence just needs the self-map about $x_{l}^{*}$ to be continuous. The Logit model (12) ensures traveler’s route choice probability is continuous with respect to route systematic disutility. In addition, if the separable link travel time function $c_{l}^{t}(x_{l}^{t},u_{l}^{t})$ adopted in the system is assumed to be continuous, then the route systematic disutility is also continuous with respect to the link flow. This contributes to the continuous self-map of $x_{l}^{*}$ and therefore the FP existence.

### 3.3 FP Uniqueness

FP uniqueness is an attractive character of traffic network model, it has been extensively studied in existing literatures ([22,27,48]). For the dynamic evolution process as considered in this paper, this issue can be analyzed by investigating the monotonicity of the self-map about $x_{l}^{*}$. In DTD system (14), the route choice probability is always non-increasing with respect to the adopted route systematic disutility $h_{r}^{*}$. However, the value of $h_{r}^{*}$ usually cannot be assured to be monotone strictly increasing with respect to the link traffic flow $x_{l}^{*}$. As a result, the FP uniqueness cannot be guaranteed. In the context of DTD dynamics, the un-uniqueness of FP or equilibrium state will give rise to irreversibility issue as discussed below.

### 3.4 FP Stability and Irreversibility

Stability is an important property of a dynamical model for its applicability in practice ([1,4,7,10,22,23,25,31,48–52]). A FP is (asymptotically) stable if from any (sufficiently close) starting state the system state tends to the fixed-point as *t* tends to infinity. If the value of the sensitivity parameter *σ* in Eq 11 is positive, then the risk attitude parameter $\rho_{od}^{t}$ is an unstable attribute against the stability of the fixed-point in dynamic system (14). In other words, any small fluctuation of network traffic flow state will cause the change of $\rho_{od}^{t}$. Obviously, the evolution of $\rho_{od}^{t}$ defined by Eq 11 is an irreversible process which means the latest risk attitude parameter $\rho_{od}^{t+1}$ will never return to the original value $\rho_{od}^{t}$ by removing the fluctuation. Under this situation, the change of route systematic disutility is also irreversible and thus the fixed-point of the dynamic system is not asymptotically stable.

On the other hand, consider the situation in which the sensitivity parameter *σ* is equal to zero-value. Substituting *σ* = 0 into Eq 11 to get $\rho_{od}^{t+1}=\rho_{od}^{t}=\cdots =\rho_{od}^{0}$, this actually assumes exogenous or constant risk attitudes for travelers. In this situation, the FP stability is also significantly affected by the parameters (including *α*, *β*, *σ* and *θ*) adopted in DTD model (14). To investigate FP stability of a deterministic, discrete-time dynamic model, a common method is to conduct a spectral analysis about the Jacobian matrix of the transition process contained in the underlying DTD evolution system. We here give a weaker stability condition by assigning sufficiently small values for parameters *α*, *β* and *θ*(*σ* = 0).

A closely related concept to FP instability is its irreversibility. When a traffic network is disturbed by some fluctuations, its flow pattern may deviate from the original FP and evolve to another new equilibrium state. A FP or equilibrium state is said to be irreversible if it cannot be restored by revoking the fluctuation. It should be pointed out that the existence of multiple equilibria is necessary for modelling irreversibility. In the proposed DTD model (14), the irreversibility phenomenon or multiple equilibria is mainly caused by the irreversible evolution process of the risk attitude parameter $\rho_{od}^{t}$ defined by Eq 11. In the existing literatures, non-separable link (or path) cost function or bounded rationality behavior model is applied to describe the irreversibility issue ([30,49]). This paper provides a new method to model this problem by considering travelers’ risk-taking behaviors.

## 4. Numerical Examples on a Test Network

**Fig 2:**
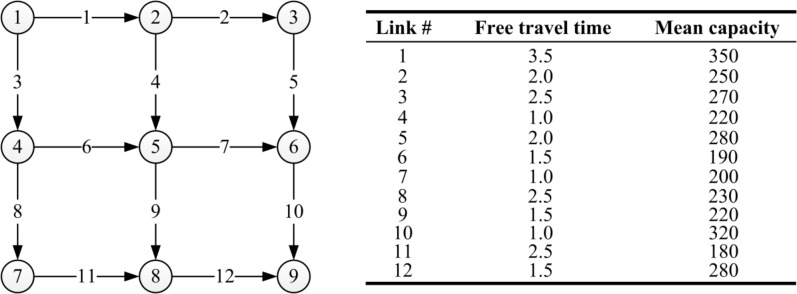
Illustration of the experiment network. The left part shows network topological structure, and the right part shows link parameters.

The proposed DTD model (14) is applied to a three-by-three grid network with nine nodes and twelve links, whose topology is illustrated in the left side of Fig 2. In the numerical example, the link travel time function is of the BPR type:

$$c_{l}(x_{l}^{t},u_{l}^{t})=c_{l}^{free}∙\left[1+0.15{\left(\frac{x_{l}^{t}}{u_{l}^{t}}\right)}^{4}\right],l=1,2,\cdots 12.$$

The values of the free flow travel time $c_{l}^{free}$ and the mean link capacity ${\dot{u}}_{l}^{t}$ are given in the right side of Fig 2. In this example, the stochastic road capacity is assumed to keep a same fluctuation range for every day, $u_{l}^{t}\in [0.8{\dot{u}}_{l}^{t},1.2{\dot{u}}_{l}^{t}]$, thus ${\underline{u}}_{l}^{t}=0.8{\dot{u}}_{l}^{t}$, ${\bar{u}}_{l}^{t}=1.2{\dot{u}}_{l}^{t}$.

One OD pair from node 1 to node 9 is considered in this network. Clearly, there are 6 routes connecting this OD pair. The daily traffic demand of this OD pair is assumed to be 500. In all test scenarios, *ρ*_max_ is set as 0.8.

### 4.1 The Effects of Parameters *α*, *β*, *σ* and *θ* on the Evolution System

**Fig 3:**
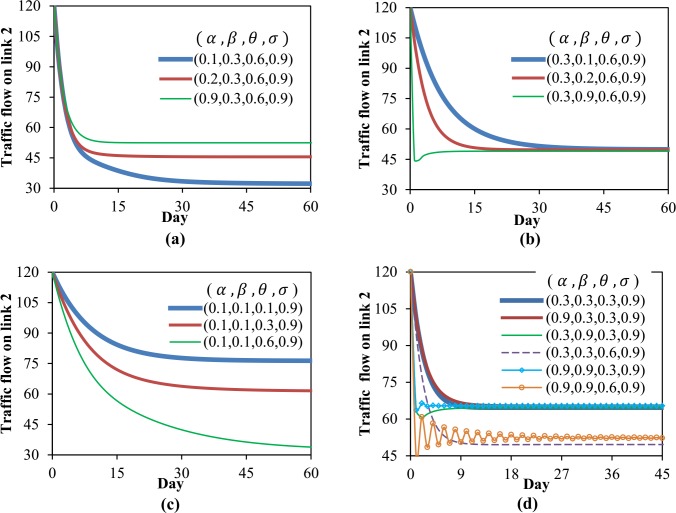
Evolution of the system with endogenous risk attitudes (*σ* = 0.9). Fig 3(a)~3(d) shows the influences of parameters *α*, *β* and *θ* on both steady state and evolution process of the dynamic system.

With different combinations of parameters *α*, *β*, *σ* and *θ*, the DTD system (14) will exhibit different evolution processes about link (or route) flow patterns. Firstly, the DTD model is executed repeatedly according to different values of *α*, *β* and *θ*, and the other parameters are kept unchanged as: $\rho_{19}^{0}=0.2$ (risk averse), *σ* = 0.9. For graph simplicity, only the flow on link 2 is adopted to illustrate the evolution of the dynamic system, as shown in Fig 3.

Fig 3(A) indicates a larger *α*-value will contribute to a faster process for the dynamic system to reach a steady state. It also demonstrates that under the condition of endogenous risk attitudes, the preference parameter *α* will significantly affect the fixed-point attractor of the evolution process, this is because *α* can influence the past travel experiences and further affect travelers’ perception of risks. In Fig 3(B), the inertial parameter *β* shows similar effect as the parameter *α*. In addition, the comparison of Fig 3(A) and 3(B) shows that, *α* has greater impact than *β* on the FP of the system. Fig 3(C) shows the dispersion parameter *θ* can also greatly influence the ultimate steady state, and a smaller *θ*-value is corresponding to a faster evolution process. In Fig 3(D), six groups of parameters *α*, *β* and *θ* are adopted to examine their combined effects on the evolution system. The result reveals the larger values of *α*, *β* and smaller value of *θ* together help to accelerate the convergence of the system, a smaller *α* or *β* or *θ* will lead to a more smooth evolution process, while a combination of larger *α*, *β* and *θ* may cause greater fluctuations.

Next, consider the situation that travelers have constant risk attitudes, this can be realized by set *σ* = 0. In this case, the parameters *α* and *β* can only influence travelers’ past experiences through affecting the evolution process of the dynamic system, but cannot affect travelers’ perception of risks since the risk attitude parameter are assumed to be constant. As a result, these two parameters will not affect the FP of the dynamic system. For the dispersion parameter *θ*, it contributes directly to the randomness of travelers’ route choice behaviors, thus it has an inherent influence on the FP no matter the risk attitude is constant or not. These analysis results can be verified by conducting a similar numerical experiment. To avoid redundancy, these numerical results are omitted here.

### 4.2 The Effects of Travelers’ Risk-taking Behaviors on Fluctuation and Evolution Convergence

Compared with the traditional DTD model, traveler’s risk-taking behavior is an additional component considered by our dynamic system to influence the whole DTD network traffic evolution process. In this subsection, a numerical experiment is designed to investigate the effects of risky route choices on fluctuation and evolution convergence of the dynamic system. The fluctuation function $\Theta^{t}=\sum_{l}\left|x_{l}^{t}-x_{l}^{t-1}\right|$ is defined to represent the aggregated link flow variation between two successive days. Obviously, when network traffic flow reach a FP or equilibrium state, Θ^*t*^ = 0.

**Fig 4:**
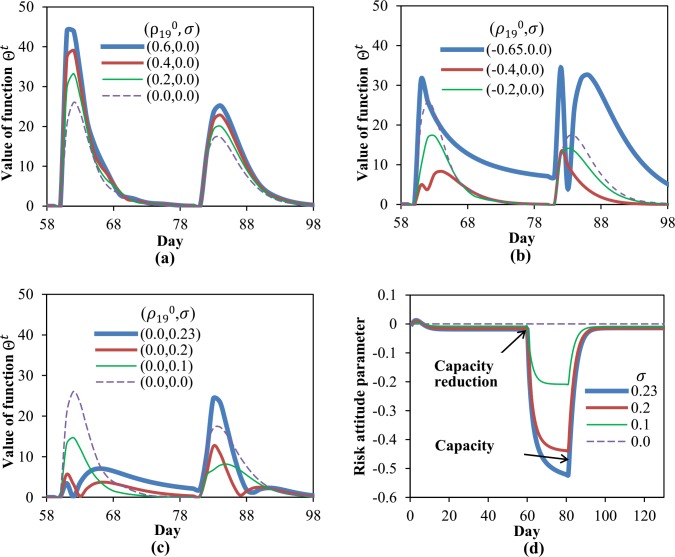
The effects of traveler risk-taking behaviors on system evolution processes. Fig 4(a) and 4(b) compare the effect difference between risk aversion and risk proneness attitudes. Fig 4(c) and 4(d) show the influences of parameter σ on both fluctuation function Θ^*t*^ and endogenous risk attitude $\rho_{19}^{t}$.

Suppose all links on the network suffer 70% mean capacity reductions on day 60 and recover to normal on day 81. Firstly, consider the situation that travelers’ risk attitudes are changeless (*σ* = 0), the DTD model (14) is executed respectively according to different initial risk attitude parameters. The other parameters are given as: *α* = *β* = *θ* = 0.3. The evolutions of Θ^*t*^ for this situation are shown in Fig 4(A) and 4(B).

Whether the link capacities decrease (occur in day 60) or increase (occur in day 81), travelers’ risk aversion route choice behaviors are found to cause some additional flow fluctuations. Fig 4(A) indicates that a more sharp risk aversion attitude (namely a larger $\rho_{19}^{0}$) leads to some greater fluctuations of the dynamic system. The observations achieved from risk aversion situation are significantly different from that appearing in risk proneness case, as shown in Fig 4(B). It can be found that in a reasonable bound, travelers’ risk proneness route choices can contribute to a smoother DTD evolution process. Beyond this bound, however, some excessive risk proneness route choices made by travelers (e.g. $\rho_{19}^{0}=-0.65$) will result in greater network flow fluctuation and slower convergence process.

By relaxing the assumption *σ* = 0, this experiment can also be used to investigate travelers’ risky behaviors with endogenous risk attitudes. Fig 4(C) presents the evolutions of Θ^*t*^ according to a same initial risk attitude ($\rho_{19}^{0}=0$) and four different sensitivity parameters *σ*. The associated risk attitude updating processes are shown in Fig 4(D).

In Fig 4(C), the effect of *σ* reflected in the capacity reduction period is found different from that appeared in capacity restoration. When link capacity reductions occur, a larger *σ* can lead to small flow fluctuation on the early days but slower convergences for later days. And when link capacities recover to normal, some greater fluctuations but faster convergences are associated to larger *σ*. This observation can be explained through studying the updating of $\rho_{19}^{t}$ presented in Fig 4(D). Obviously, link capacity reductions intensify flow congestions and further cause travel losing perceived by travelers, this results in the evolution of risk attitude towards proneness, namely the decreasing of $\rho_{19}^{t}$. After capacity reductions, a large *σ* permits $\rho_{19}^{t}$ to reach a preferable value (e.g. -0.4) quickly, which benefits the smooth-evolution of the dynamic system on the early days. On the subsequent days, however, a large *σ* may also push $\rho_{19}^{t}$ to reach a too small value which reflecting excessive risk proneness behaviors, this certainly give rise to greater fluctuations and slower convergences. For the situation of capacity restoration, a similar analysis can be applied to explain the result appearing in Fig 4(C).

### 4.3 The Effects of Travelers’ Risk-taking Behaviors on FP Stability and Irreversibility

As introduced in the previous Subsection 3.4, the fixed-point stability or reversibility can be regarded as an indicator evaluating road network resilience against fluctuations. In this numerical experiment, some reductions of mean link capacity are still introduced into the DTD model to reflect the external disturbances of the traffic network. A positive-value and a zero-value are respectively assigned to the sensitivity parameter *σ* to reflect two types of travelers’ risk attitude evolution schema. The initial risk attitude parameter $\rho_{19}^{0}$ is set as 0.3 (risk averse).

**Fig 5:**
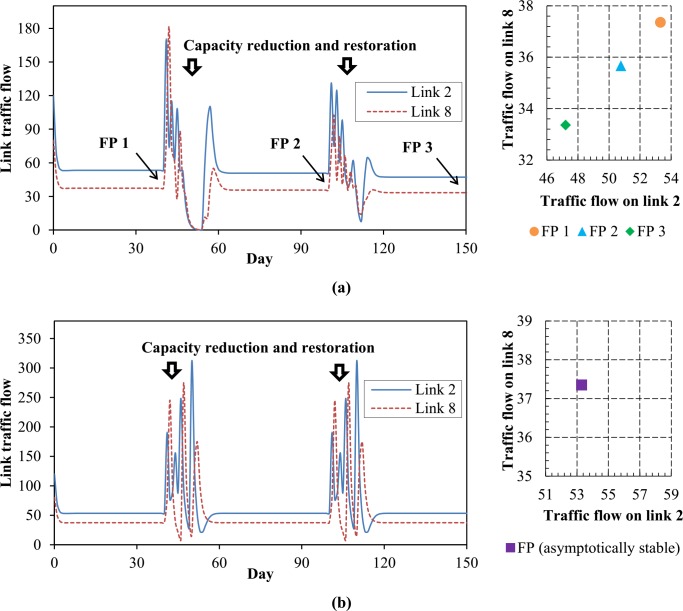
Comparison of effects between two different risk attitude evolution schemas. Fig 5(a) corresponds to the case of endogenous risk attitudes, and Fig 5(b) corresponds to the case of exogenous risk attitudes.

On two time periods respectively from the day 40 to 50 and the day 100 to 110, all the links are assumed to suffer 50% mean capacity reductions, and outside of these two periods, these link capacities all restore to their original values. Throughout the second experiment, the following model parameters are used and kept fixed: *α* = *β* = *θ* = 0.6. Under this situation, the DTD model (14) is executed respectively according to different risk attitude evolution schemas. The link flow evolution trajectories and the corresponding FPs are shown in Fig 5.

Fig 5(A) indicates that when travelers’ risk attitudes are endogenous, their route choice behaviors will cause the FP instability. That is, any fluctuation of link capacity will give rise to the deviation from the original FP, and drive the dynamic system to reach a new equilibrium state but not the original one even though the changed link capacities are revoked, this in fact corresponds to the irreversibility. In Fig 5(B), however, capacity reduction and restoration are found only to cause some fluctuations on the evolution process but not to change the ultimate equilibrium state, which means, under the situation of exogenous or constant risk attitudes, FP of the DTD model is stable. Note that this stability is only satisfied in the attraction domain of the FP. This is because the route systematic disutility function usually cannot be assured to be monotone strictly increasing with respect to the link traffic flow. And as a result, the FP uniqueness cannot be guaranteed. Therefore the FP only meets, strictly speaking, the asymptotically stability condition in this situation.

Due to the short of empirical data, we conducted numerical experiments only on a simple grid network in this section. A real transport network is usually not so regular and its topological structure is more complicated. It is meaningful to test the proposed model on a large-scale real transport network. Meanwhile, a real transport network usually contains multiple travel OD pairs and a larger number of links. This means the route-based flow assignment approach, which is defined by the Logit model in this paper, may become invalid since the number of feasible routes will increase exponentially. Therefore, it is also necessary to design a more effective method for executing the proposed DTD model under the situation of real transport network. We leave these researches to our future work.

Experimental data appeared in the above-mentioned figures can be achieved directly by running the program source code of the numerical experiment. In this paper, the experiment program is written by Visual C and executed on a T2250 CPU (2.50Ghz). The Experimental data are saved on “S1 Information. Experimental data for Figs 3, 4 and 5.” The program source code are saved on “S2 Information. Program source code for Figs 3, 4 and 5.”

## 5. Conclusion and Future Work

This paper aims to model DTD flow dynamics on degradable transport network by considering both travelers’ study of uncertain travel time and travelers’ choice of risky routes. The notion of variation range is adopted to represent travelers’ perceptions of travel time uncertainty. In addition, an endogenous risk attitude evolution schema is adopted to reflect the change of traveler’s risk attitude in the context of DTD traffic dynamics. The uncertain route travel time and the risk attitude parameter are both integrated into a unified systematic disutility function to reflect travelers’ perception of route attractiveness. These route disability values are substituted into a Logit model to describe travelers’ stochastic route choice behaviors. This paper also makes some effects to investigate several mathematical properties implied in the proposed DTD model. Numerical results obtained from a test network verify that some moderate risk proneness route choices made by travelers are beneficial to a smoother DTD evolution process, while risk aversion behaviors as well as excessive risk proneness route choices will both give rise to greater fluctuation and slower convergence of the dynamic system. In addition, when travelers’ risk attitudes are endogenous, their DTD dynamic route adjustment behaviors will indeed lead to FP instability and irreversibility. Although we focus on transport network in this study, our research may also benefit other relevant fields such as traffic dynamics on complex networks (e.g. [53–56]).

For the proposed DTD model, quite a number of parameters are adopted to influence its dynamic evolution trajectory. Calibration of these parameters is worth of further research effort. In this paper, a simple update Eq 11 is formulated to reflect the endogenous risk attitude evolution schema, but it may not conform to the actual case. Therefore, it is meaningful to design more realistic formulations reflecting travelers’ risk attitude changes in the future work. Given that the dynamic model may have multiple equilibria, it is also interesting to analytically derive the sufficient condition that assures the asymptotically stability of each fixed point.

## Supporting Information

S1 Information Experimental data for Figs 3, 4 and 5. (XLSX)

S2 Information The program source code for Figs 3, 4 and 5. (TXT)
